# Exploiting Language Models to Classify Events from Twitter

**DOI:** 10.1155/2015/401024

**Published:** 2015-09-14

**Authors:** Duc-Thuan Vo, Vo Thuan Hai, Cheol-Young Ock

**Affiliations:** ^1^School of Electrical Engineering, University of Ulsan, 93 Daehak-ro, Nam-gu, Ulsan 680-749, Republic of Korea; ^2^Soongsil University, 369 Sangdo-ro, Dongjak-gu, Seoul 156-743, Republic of Korea

## Abstract

Classifying events is challenging in Twitter because tweets texts have a large amount of temporal data with a lot of noise and various kinds of topics. In this paper, we propose a method to classify events from Twitter. We firstly find the distinguishing terms between tweets in events and measure their similarities with learning language models such as ConceptNet and a latent Dirichlet allocation method for selectional preferences (LDA-SP), which have been widely studied based on large text corpora within computational linguistic relations. The relationship of term words in tweets will be discovered by checking them under each model. We then proposed a method to compute the similarity between tweets based on tweets' features including common term words and relationships among their distinguishing term words. It will be explicit and convenient for applying to k-nearest neighbor techniques for classification. We carefully applied experiments on the Edinburgh Twitter Corpus to show that our method achieves competitive results for classifying events.

## 1. Introduction

Twitter (https://twitter.com/) is a social networking application that allows people to microblog about a broad range of topics. Users of Twitter post short text, called “tweets” (about 140 characters), on a variety of topics as news events and pop culture, to mundane daily events and spam. Recently, Twitter has grown over 200 million active users producing over 200 million tweets per day. Twitter is a popular microblogging and social networking service that presents many opportunities for researches in natural language processing (NLP) and machine learning [1–6]. Locke and Martin [5] and Liu et al. [4] train a classifier to recognized entities based on annotated Twitter data for Named Entity Recognition (NER). Some research has explored Part of Speech (PoS) tagging [3], geographical variation in language found on Twitter [2], modeling informal conversations [1], and also applying NLP techniques to help crisis workers with the flood of information following natural disasters [6]. Benson et al. [7] applied distant supervision to train a relation extractor to recognize artists and venues mentioned within tweets of users who list their location.

Classifying events in Twitter is a difficult task that focuses on the automatic identification and classification of various types of events in tweet texts. In Twitter, events are topics that often draw public attention, for example, football matches or natural disasters. Several approaches have been proposed to classify events for detection such as wave analysis [8, 9], topic model approach based on latent Dirichlet allocation [10], hierarchical Dirichlet processes [11], and text classification and clustering [12]. Kireyev et al. [8] explored the use of topics models for analysis of disaster-related Twitter data. Sakaki et al. [12] investigated the real-time interaction of events such as earthquakes in Twitter and proposed an algorithm to monitor tweets and to detect target events. However, existing approaches encounter failures from in either latent topics detection or analyzing terms relationships. Because topic model techniques [13–15] have only focused on how to list set of relevant words into a group (called topic) it is missed on analyzing relations between topics. Considering tweets have been discussed in two events shown in Table 1, we are easy to recognize that T_1_ and T_2_ are discussed in event 1 and T_4_ and T_5_ are discussed in event 2. However, if using topic models the system will group T_1_, T_2_, and T_3_ in the same event category even T_3_ does not belong to the event because set of relation words as <“*passed away*,” “*dead*,” “*died*”> in these tweets is in the same topic model. Likewise, T_6_ will be grouped into event 2 with T_4_ and T_5_ together even if T_6_ does not belong to this event because sets of relation words as <“*plane*,” “*crash*,” “*helicopter*”>, <“*Russia*,” “*KHL team*,” “*Lokomotiv*,” “*hockey*”>, and <“*kills*”, “*dead*”> in these tweets are within the same topic models, respectively. Due to limitations in using topic models, we therefore propose the method to exploit language models having relations reference to not only analyze topics but also analyze relatedness of event in tweets to overcome these problems.

In this paper, we investigate the use of generative and discriminate models for identifying the relationship of objects in tweets that describe one or more instance of a specified event type. We adapt language modeling approaches that capture how descriptions of event instances in text are likely to be generated. Our method will find the distinguishing term words between tweets and examining them with a series of relationships, extracted by language models such as ConceptNet [16] and LDA-SP [17]. These language models have been widely studied based on large text corpora within computational linguistic relations. Hence the relationship among distinguishing terms and common terms between tweets becomes clear to measure their similarity by examining them under each model. Measuring similarity between tweets is explicit and convenient to apply it in the classifier algorithms, such as SVM and k-nearest neighbor (*k*NN), to classify events in Twitter.

The rest of this paper is structured as follows.  Section 2 presents related work that refers to research on event detection. In Section 3, we discuss exploiting language models. In addition, we present a method to calculate the similarity between tweets for event classification. In the next following section, experiments that are applied to the Edinburgh Twitter Corpus for event classification are presented and discussed.  Section 5 ends with conclusions and future work.

## 2. Related Work

Several applications have detected events in Web to apply to weblogs [18–20], news stories [21, 22], or scientific journal collections [23]. Glance et al. [19] presented the application of data mining, information extraction, and NLP algorithms for event detection across a subset of approximately 100,000 weblogs. They implemented a trend searching system that provides a way to estimate the relative buzz of word of mouth for given topics over time. Nallapati et al. [22] attempted to capture the rich structure of events and their dependencies on a news topic through event models by recognizing events and their dependencies on event threading. Besides the standard word for based features, their approaches took into account novel features such as the temporal locality of stories for event recognition. Besides that, some researches [24–27] have analyzed social network to search or detect emergency events on the internet. Dai et al. [25] presented a cycle model to describe the internet spreading process of emergency events which applied the Tobit model by analyzing social psychological impacts. Hu et al. [27] analyzed historical attributes then combined with HowNet polarity and sentiment words on microblog which has network information transmission of social emergency events. And, they then provided the important guidance in the analysis of microblog information dissemination that has relatedness with social emergency events on internet. Meanwhile, Dai et al. [24] proposed a method to search the shortest paths of emergency events through IBF algorithm by analyzing social network.

Some research has focused on summarizing Twitter posts for detecting events [28–31]. Harabagiu and Hickl [28] focused on the summarization of microblog posts relating to complex world events. To summarize, they captured event structure information from tweets and user behavior information relevant to a topic. Takamura et al. [31] summarized Japanese Twitter posts on soccer games during the time when people provide comments and expressed opinions on the timeline of a game's progress. They represented user actions in terms of retweets, responses, and quoted tweets. In particular, Sharifi et al. [30] detected events in Twitter by summarizing trending topics using a collection of a large number of posts on a topic. They created summaries in various ways and evaluate those using metrics for automatic summary evaluation.

Recently, several approaches have been proposed to detect events from tweets using topic model approach [8, 10, 12]. Kireyev et al. [8] explored the use of topic models for the analysis of disaster-related Twitter data. Becker et al. [32] and Popescu et al. [33] investigate discovering clusters of related words or tweets which correspond to events in progress. Sakaki et al. [12] investigated the real-time interaction of events in Twitter such as earthquakes and propose an algorithm to monitor tweets and to detect a target event. Diao et al. [10] attempted to find topics with bursty patterns on microblogs; they proposed a topic model that simultaneously captures two observations such as posts published around the same time and posts published by the same user. However, existing approaches have still met with failure in either latent topic detection or analyzing relationship terms, because tweets messages usually contain very limited common words in topics. Therefore, in this paper we propose a method to discover the relationship of objects in tweets by exploiting language models used to compare each of the snippets indirectly for classifying events in Twitter.

## 3. Exploiting Language Models to Classify Events

**Figure 1:**
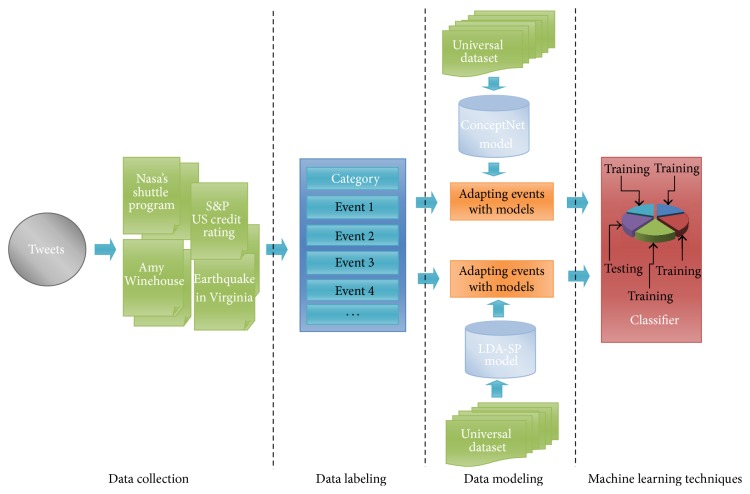
Proposed method.

In this paper, we investigate the use of generative and discriminate models for identifying the relationship among objects in tweets that describe one or more instances of a specified event type. We adapt language modeling approaches that capture how descriptions of event instances in text are likely to be generated. We use language models to select plausible relationships between term words in tweets such as the relationship of “Object-Object” or “Object-relation-Object,” which aim to detect the relatedness of an event in tweets. We assume that the data collection of language models contains suitable knowledge on the relationships among term words to discover the elemental relationship among tweets with a statistical analysis to classify events. We explore two types of language models that have obtained high correlation with human judgment such as ConceptNet and LDA-SP. These models are used for calculating the similarity of a pairwise of tweets for detecting events. The relationship between the discriminate term words of the tweets will be discovered by checking their relatedness under pairs of relations. In addition, the similarity between tweets is computed based on their common term words and the relationship between their discriminate term words. It is intuitive and convenient to apply it in classifier algorithms to classify events in Twitter. The general proposed method consists of four stages as (1) data collection, (2) labeling stage, (3) data modeling, and (4) machine learning shown in Figure 1. Stages 1 and 2 will be discussed in Section 4.1; stage 3 will discussed in Section 3; and state 4 will be discussed in Sections 3.3 and 4.2.

### 3.1. ConceptNet Model

To model the “Object-Object” relationships in tweets, we consider the ConceptNet [16] model. It is a large semantic graph containing concepts and the relations between them. It includes everyday basic, cultural, and scientific knowledge, which has been automatically, extracted from the internet using predefined rules. In this work, we use the most current version ConceptNet 5. As it is mined from free text using rules, the database has uncontrolled vocabulary and contains many false/nonsense statements. ConceptNet contains 24 relations with over 11 million pairs of relation. For example, “Nasa is located in United States” is presented as AtLocation (“Nasa”, “United States”) in ConceptNet model.  Table 2(a) shows list of 24 relations, and Table 2(b) shows samples of four relations as MadedOf, AtLocation, MotivedbyGoad, and RecievesAction. Speer and Havasi [16] provide more details of the model in their paper. We first examine all relations in the ConceptNet 5 database (http://conceptnet5.media.mit.edu/) and define which are relevant to relations in target events by keywords matching (in experiments) to extract relations.

### 3.2. LDA-SP Model

**Figure 2:**
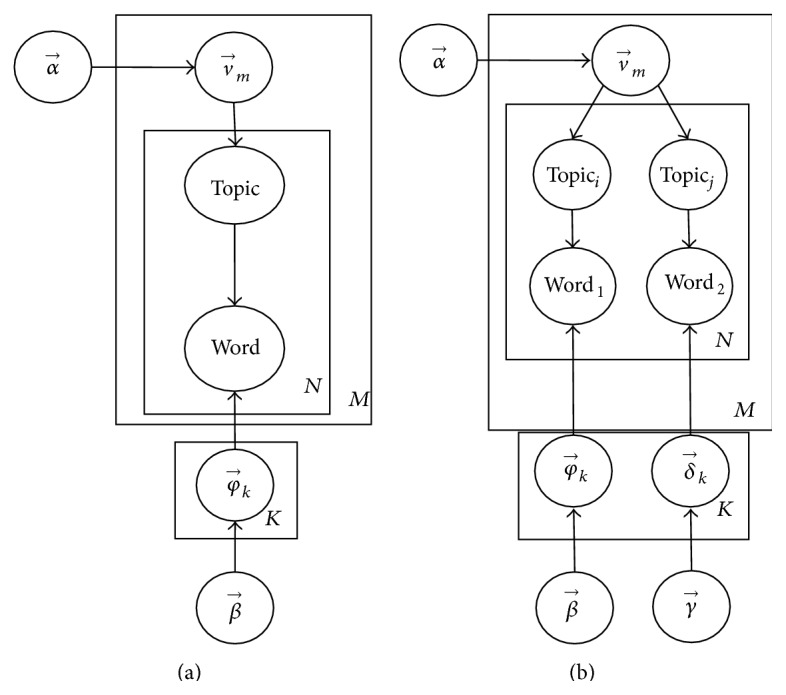
Graphical model of LDA model (a) versus LDA-SP (b).

To model the “Object-relation-Object” relationships in tweets, we adapt the LDA-SP model [17], which has been used for the selectional preference task in order to obtain the conditional probabilities of two objects in a relation. In particular, the LDA-SP, using LinkLDA [34], is an extension of latent Dirichlet allocation (LDA) [13] which simultaneously models two sets of distributions for each topic. The generative graphical model of LDA versus LDA-SP is depicted in Figure 2. In LDA-SP, they presented a series of topic models, at which objects belonged to them, for the task of computing selectional preferences. These models vary in terms of independence between* Topic*_*i*_ and* Topic*_*j*_ that is assumed. These two sets represent the two arguments for the relation *R*(*Topic*_*i*_, *Topic*_*j*_). Each topic contains a list of relation words. Each relation,* R*, is generated by picking up over the same distribution, which keeps two different topics,* Topic*_*i*_ and* Topic*_*j*_, sharing the same relation (Figure 2(b)). The LDA-SP is able to capture information about the pairs of topics that commonly cooccur. To model the relations with LDA-SP, we also follow the data preparation in [21], which was automatically extracted by TextRunner [35] from 500 million Web pages. This resulted in a vocabulary of about 32,000 noun phrases, a set of about 2.4 million tuples with 601 topics in our generalization corpus. Some samples of topics extracted through LDA-SP are illustrated in Table 3.

### 3.3. Similarity Measures in Tweets

Classifying events in tweets from Twitter is a very challenging task because a very few words cooccur in tweets. Intuitively, the problem can be solved by exploring the relationships between tweets well; the intrinsic relationship among words may be discovered with a thesaurus. Hence, we present a method to discover the intrinsic relationships between objects based on statistical analysis of language models and then gain the similarity between tweets accordingly. We consider two types of relationships in tweets such as “Object-Object” and “Object-relation-Object.”

**Figure 3:**
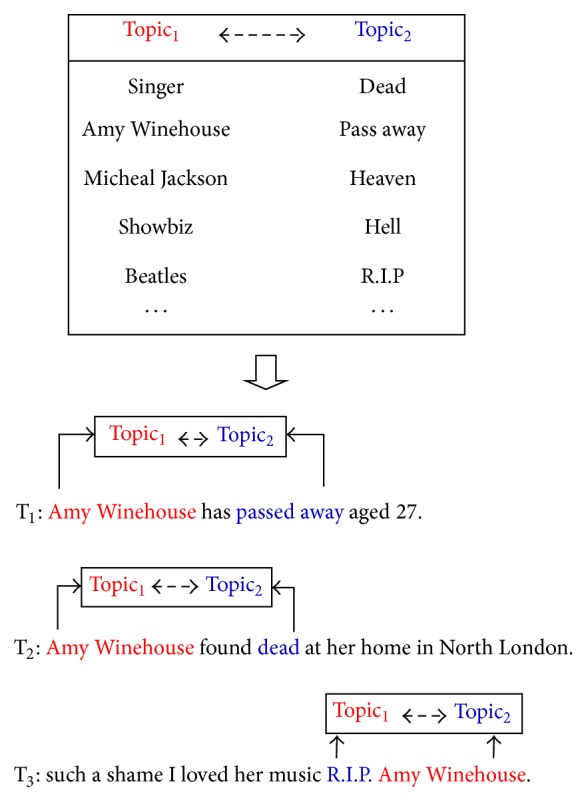
Relationship “*Topic*_*1*_ *-Topic*_*2*_” in tweets of event “Death of Amy Winehouse.”

“*Object-Object*”. The event “*Death of Amy Winehouse*” is posted in tweets T_1_, T_2_, and T_3_ shown in Figure 3. Traditional methods can only find one cooccurring term, “*Amy Winehouse*,” in the tweets after removing stop words. However, if we analyze and compare the relatedness between the pairs <“*Singer*”-“*Amy Winehouse*”>, <“*Amy Winehouse*”-“*passed away*”> and <“*Amy Winehouse*”-“*dead*”>, and <“*Amy Winehouse*”-“*R.I.P.*”>, closer relationships will be exposed: “*Object-Object*” as “*Topic*_*1*_ *-Topic*_*2*_” where a set of terms {“*Singer*”; “*Amy Winehouse*”} is in* Topic*_*1*_ and a set of terms {“*death*”, “*passed away*”, “*R.I.P.*”} is in* Topic*_*2*_.

**Figure 4:**
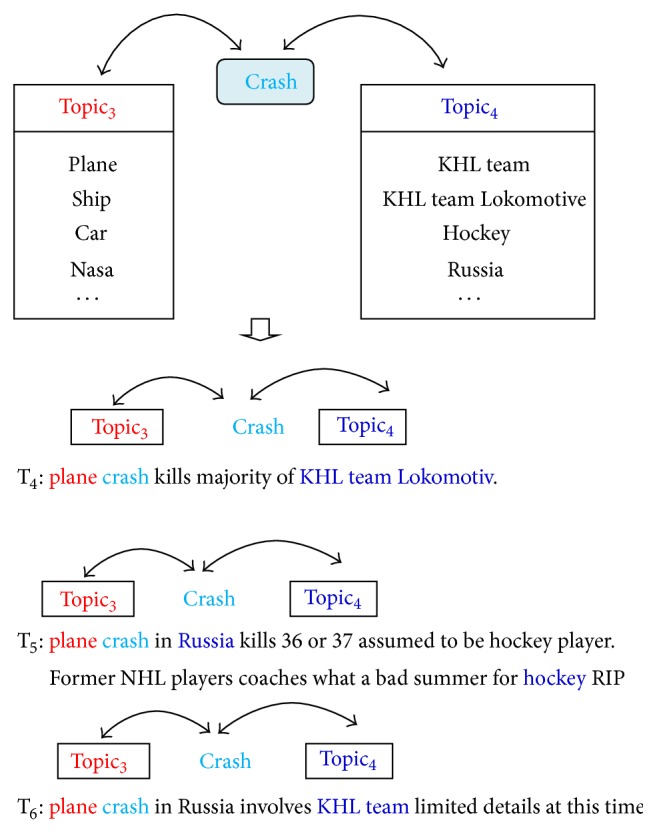
Relationship “*Topic*_*3*_ *-relation-Topic*_*4*_” in tweets of event “plane carrying Russian hockey team Lokomotiv crashes.”

“*Object-Relation-Object*.” The event “plane carrying Russian hockey team Lokomotiv crashes” is posted in T_4_, T_5_, and T_6_ shown in Figure 4. We can discover the relationship between “Object-relation-Object” such as <“*Plane*”-“*crash*”-“*KHL team Lokomotiv*”>, <“*Plane*”-“*crash*”-“*Russia*”>, and <“*Plan*”-“*crash*”-“*KHL team*”>. This also exhibits the closer relationships “*Object-relation-Object*” as “*Topic*_*3*_ *-crash-Topic*_*4*_” where the term {“*plane*”} belongs to* Topic*_*3*_ and a set of terms {“*russia*”, “*khl team lokomotiv*”, “*hockey*”, “*khl team*”} belongs to* Topic*_*4*_.

Our method extracts relation tuples from language models such as ConceptNet and LDA-SP. We treat all tweets from Twitter that are contained in the collection equally and then perform to match models of tuples generated from ConceptNet and LDA-SP with them. Hence, if we can discover relation tuples as “third-party” for both tweets and calculate the similarity between the two tweets by comparing the distinguishing term words with these tuples, we may find the real relationship underlying the two tweets. We assume that the data collection language models contain sufficient knowledge about the relationships among term words, from which we can find the elemental relationship among tweets.

For computing similarity between tweets, we derive a set of relations, *R*_*i*_ = (*object*_*m*_, *object*_*n*_) matched from language models and tweets combining with Bag-of-Words. Considering two original tweets, *d*_1_ and *d*_2_, in data collection *D*, we check with *object*_*m*_, *object*_*n*_ existing in each tweet which match with relation tuples *R*_*i*_ = (*object*_*m*_, *object*_*n*_) extracted from ConceptNet model. In using LDA-SP, we exam not only relations but also *object*_*m*_, *object*_*n*_ existing in each tweet and then match them with relation tuples *R*_*i*_ = (*object*_*m*_, *object*_*n*_) generated from LDA-SP. We then replace matched objects in tweets by relation tuples from language models. Thus, the relationship between the distinguishing terms of the tweets can be discovered by examining their relatedness under pairs of relations by “third-party.” We consider calculating the similarity between two tweets based on their common terms and the relationship between their distinguishing terms. To calculate the similarity between two tweets in an event category, we represent them as vectors:

(1) $$\begin{array}{c} d_{1}=\left(w_{1},w_{2},\ldots ,w_{n}\right)\\ d_{2}=\left(w_{1},w_{2},\ldots ,w_{n}\right), \end{array}$$

where *w*_*i*_ is the weight of the *i*th feature in the vector of *d*_*j*_ and is defined by the tf-*i*df measure as follows:

(2) $$\begin{array}{c} w_{i}=\text{t}{\text{f}}_{ij}\times \text{lo}{\text{g}}_{2}\left(\frac{M}{\text{d}{\text{f}}_{j}}\right), \end{array}$$

where *M* is the total number of documents in the collection, df_*j*_ is the document frequency, that is, the number of documents in which term *w*_*i*_ occurs, tf_*ij*_ is the term frequency of term *w*_*i*_ in document *d*_*j*_, and tf_*ij*_ is simply the number of occurrences of term *w*_*i*_ in document *d*_*j*_.

With the relationship between the two distinguishing term words on a diversity of assigned model tuples, we can calculate the similarity of vectors *d*_1_ and *d*_2_ with the cosine method shown in

(3) $$\begin{array}{c} \text{sim}\left(d_{1},d_{2}\right)=\cos\left(d_{1},d_{2}\right)=\frac{d_{1}\cdot d_{2}}{\left|d_{1}\right|\left|d_{2}\right|}=\frac{\sum_{i=n}w_{i1}w_{i2}}{\sqrt{\sum_{i=n}w_{i1}^{2}}\sqrt{\sum_{i=n}w_{i2}^{2}}}. \end{array}$$

For classifying events from tweets, many classifiers first need to calculate the similarity between tweets.* k*NN is one of the best methods of similarity calculation and selection of a proper number of neighbors. Therefore, it is intuitive and convenient to apply similarity calculation between tweets to* k*NN for classifying events. If our proposed method can calculate the similarity among tweets more accuracy, the* k*NN will select more appropriate neighbors for a test case and the classification performance of* k*NN will be higher than original tf-*i*df, since the performance of* k*NN based on the similarity measuring method outperforms other methods with tf-*i*df measure. We conclude that the proposed method is more effective on calculating tweets similarity to classify events. The result will be discussed in more detail in experimentation section.

## 4. Experimentation

### 4.1. Experimental Datasets and Evaluation Measures

We have conducted experiments on the Edinburgh Twitter Corpus [36], a collection of events in Twitter, for event classification. The corpus contains 3034 tweet IDs spread into 27 event categories. Currently, some tweets in the dataset are deleted or lost from Twitter. We developed a tool using Twitter API (http://twitter4j.org) to collected documents including tweets, retweets, responses, and quoted tweets; we then filtered documents to guarantee that each event category contains at least 70 tweets. After the removal of noise and stop words, each word is stemmed into its root form.  Table 4 shows the rest of nine significant event categories with checked mark for experiments as event 1, event 6, event 7, event 9, event 13, event 14, event 15, event 16, and event 21.

In this study, experiments are evaluated based on the precision, recall, and *F*-measure with our proposed method. The precision, recall and *F*-Measure are the evaluation metrics often used to rate the information retrieval system's performance. Precision is the number of correct results divided by the total number of returned responses; recall is the number of correct results divided by the number of results that should have been returned and *F*-measure is used to balance between the recall and precision as follows:

(4) $$\begin{array}{c} \text{Precision}=\frac{\text{number}\_\text{of}\_\text{correct}\_\text{responses}}{\text{number}\_\text{of}\_\text{responses}},\\ \text{Recall}=\frac{\text{number}\_\text{of}\_\text{correct}\_\text{responses}}{\text{number}\_\text{of}\_\text{corrects}},\\ F\text{-measure}=\frac{2\times \text{recall}\times \text{precision}}{\text{recall}+\text{precision}}. \end{array}$$

### 4.2. Experiments and Comparison

Checking similarity between tweets before experiments, we select some samples of tweets from experimental datasets as shown in Table 1. We used the tf-*i*df combined with the similarity functions to compare performance before and after using language models. Note that T_1_ and T_2_ were discussed in the same event; T_4_ and T_5_ were also discussed in the same event. And two pairs of tweets are, respectively, to calculate similarity with stop words removal. The result depicted in Table 5 shows that the tweets using ConceptNet and LDA-SP increase the similarity of questions from the same category. Moreover, if the tweets did not belong to target event like T_3_ and T_6_, the method will reduce the similarity measure that helps system performance of classifying efficiently.

To classify events, 70% of the tweets for each category are randomly selected for training, and the rest is for testing. In our experiments, we compare the performance of four classifiers implemented as follows: (1) baseline* k*NN (without language model); (2) baseline SVM; and the* k*NN method combining our proposed methods (3)* k*NN-M1 (*k*NN with language model ConceptNet) and (4)* k*NN-M2 (*k*NN with language model LDA-SP). The SVM is also constructed using the tf-*i*df method to weight each vector component of the tweet and is used as second baseline for comparison with our proposed methods. We chose SVM because of a powerful and robust method for text classification [37–39]. The evaluation follows 5-fold cross validation schema.  Table 6 shows the performance results applied to 7 categories of events from Twitter. The bold numbers show the best *F*-measure of each event in four methods. For instance, the system obtained the highest *F*-measure of 85.3% in event 1 with method* k*NN-M2. Method* k*NN-M1 yielded better *F*-measure results in most of the event categories: event 6, event 7, event 9, event 14, event 15, and event 16. And, method* k*NN-M2 achieved better *F*-measure result in three categories: event 1, event 13, and event 21.

**Figure 5:**
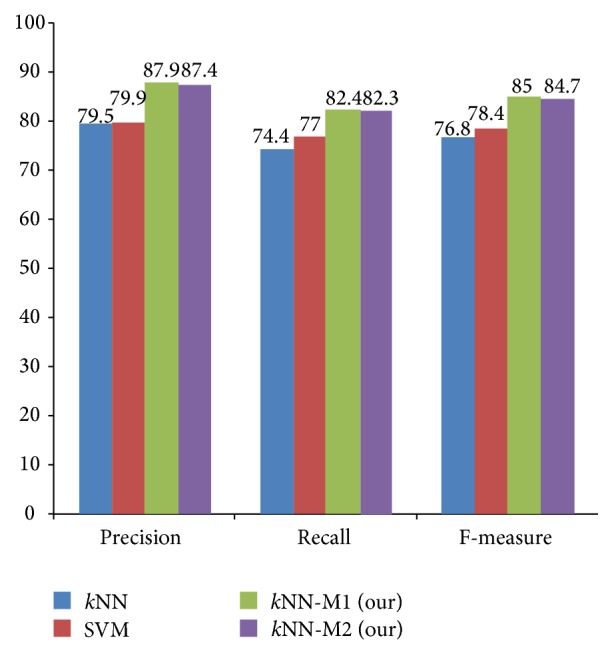
Overall performance comparisons.

The overall performance comparison is presented in Figure 5. We can see that the performance of* k*NN-M1 outperforms* k*NN-M2, SVM, and* k*NN. Both of our proposed methods are also higher than the baselines,* k*NN and SVM, in most of the performance metrics. In the overall results,* k*NN-M1,* k*NN-M2, SVM, and* k*NN obtained an *F*-measure of 85%, 84.7%, 78.4%, and 76.8%, respectively.

### 4.3. Discussions

We believe that effective performance of our proposed methods is result of the following reasons.

First, noise and exclamative and repeated texts usually occur in the tweets of each event. The following are examples of such tweets. T_1_: “*Sad day Sky sources now confirming Amy Winehouse is dead A musical legend who died way too young in my opinion*,” T_2_: “*Amy Winehouse found dead in her London flat according to sky news*,” and T_3_: “*Hmm…omg…gruuu Amy Winehouse is dead not totally surprised though ohhh*.” We can observe that {“*Amy Winehouse*”; “*dead*”} is repeated text, {“*gruuu*”; “*ohhh*”} is noise text, and {“*Hmm*”; “*omg*”} is exclamative text. The repeated text will result in a positive value in the similarity measure; however, noise and exclamative texts will result in a negative value in the similarity measure. For preprocessing, stop words had been removed by a defined list of stop words automatically. However, we had checked and revised noise texts manually if they do not belong to list of stop words. For example, a lot of words “deaddddd” will be revised into “dead,” or {“RIP,” “R I P”} will be revised into “R.I.P.”

The second reason we believe our method had effective performance is that quality universal datasets are used to build language models. In this study, more than five billion relation records extracted from Concept are used to build the models. In addition, models from LDA-SP are built by extracting 2.4 million tuples of relations and 601 topics. Furthermore, ConceptNet is a graphical relationship model which uses predefined rules. However, LDA-SP still has some errors [17] in computing word statistics. In the experiment results, performance of ConceptNet is better than LDA-SP.

The third reason believed to be behind our method's effective performance is that the models extracted from LDA-SP are intensely analyzed compared to ConceptNet for relationship. However ConceptNet obtained better performance results. Texts from tweets are incomplete sentences that result in failures in grammar parsing for analyzing relation. We did not include grammar parsing for analyzing tweets based on LDA-SP model. Therefore, ConceptNet exhibits a better performance for classifying events from Twitter than LDA-SP.

## 5. Conclusion and Future Work

We have presented methods to classify events from Twitter. We first find the distinguishing terms between tweets in events and calculate their similarity with learning language models: LDA-SP and ConceptNet. Next, we discover the relationship between the distinguishing terms of the tweets by examining them under each model. Then, we calculate the similarity between two tweets based on their common terms and the relationship between their distinguishing terms. The outcomes make it convenient to apply* k*NN techniques to classify events in Twitter. As a result, our approach obtained better performance results with both ConceptNet and LDA-SP than other methods.

Regarding future work, the research has been suggested with attractive aspects to improve as follows. First, this approach can be considered for future work, including it with a larger corpus and experimenting with other event types. Second, we will continue to investigate how to apply grammar parsing in tweets so that we can analyze deeply relationships to serve for classifying events. Finally, the research can be applied unsupervised learning with semantic similarity models as pointwise mutual information (PMI) [40, 41] and latent semantic analysis (LSA) [42, 43].

## Figures and Tables

**Table 1:** Some samples of discussed tweets in two events.

Category	Tweets	Relatedness with event
Event 1	T_1_: Amy Winehouse has passed away aged 27.	Yes
	T_2_: Amy Winehouse found dead at her home in North London.	Yes
	T_3_: Nelson Mandela, who led the peaceful transition from white-only rule, has died aged 95.	No

Event 2	T_4_: plane crash kills majority of KHL team Lokomotiv.	Yes
	T_5_: plane crash in Russia kills 36 or 37 assumed to be hockey player.	Yes
	T_6_: plane crash, helicopter, was in Moscow with 2 dead.	No

**(a):** 

**MotivatedByGoal; CausesDesire; WordNet/ParticipleOf; MemberOf; HasA; NotDesires; UsedFor; AtLocation; Entails; DefinedAs; InstanceOf; HasPainIntensity; ReceivesAction; SimilarTo; RelatedTo; NotHasProperty; PartOf; HasLastSubevent; TranslationOf; HasProperty; NotHasA; CapableOf; WordNet/adverbPertainsTo; NotCapableOf; LocationOfAction; SimilarSize; HasPainCharater; HasContext; NotMadeOf; HasFirstSubevent; SymbolOf; LocatedNear; NotUsedFor; ObstructedBy; Desires; DerivedFrom; HasSubevent; MadeOf; Antonym; CreatedBy; Attribute; DesireOf; IsA; Causes**	

**(b):** 

MadeOf		AtLocation		MotivatedByGoal		ReceivesAction	
Atomic bomb	Uranium	Nasa	United states	Fight war	Freedom	Bacteria	Kill
Computer	Silicon	Alcoa	Pittsburgh	Get drunk	Forget life	Army tank	Warfare
Gas	Oil	Tv channel	Russia	Pen	Write letter	Bread	Cook
Song	Music	Aozora bank	Japan	Join army	Defend country	Candle	Burn for light
Person	Live cell	Apartheid	Mall	Kill	Hate someone	Tomato	Squash
Light	Energy	Golden gate	Bridge	Live life	Pleasure	Tobacco	Chew
Carton	Wax paper	Art	Gallery	Sing	Performance	Supply	Store
Chocolate	Cocoa bean	Audience	Theatre	Socialize	Be popular	Ruby	Polish
Telephone	Electronics	Crab	Coastal area	Study	Concentrate	Money	Loan
Window	Glass	Handgun	Army	Visit museum	See history	Life	Save
⋯	⋯	⋯	⋯	⋯	⋯	⋯	⋯

**(a):** 

**Topic 2: **driver; civilians; soldiers; motorcyclist; teenager; thomas; policeman; us soldier; smith; george; motoris; father; …	
**Topic 19: **china; business line; japan; government; israel; judge; india; iran; russia; democrats; court; lawmakers; …	
**Topic 106: **myspacetv videos; the trail; leaves; moses; the curtain; stones; santa; flowers; victim; posters; stars; flames; …	
**Topic 114: **britney spears; paris hilton; angelina jolie; tom cruise; the actress; lindsay lohan; amy winehouse; singer; …	
**Topic 116: **fire; violence; the war; the storm; fighting; katrina; explosion; tornado; earthquake; civil war; dead; heaven; …	
**Topic 171: **john; david; mark; mike; steve; bill; michael; peter; scott; smith; johnson; brown; executive; robert; jeff; brian; …	
**Topic 251: **police; group; team; company; day; year; case; report; miller; officials; king; wilson; story; news; friday; …	
**Topic 286: **article; report; author; court; bible; story; letter; paul; reuters; researchers; statement; respondents; …	
**Topic 390: **car; train; vehicle; bus; fingers; truck; boat; plane; river; route; traffic; driver; aircraft; train; track; bike; …	
**Topic 428: **airplane; aircraft; pilot; sparks; birds; crew; terrorists; nasa; people; passengers; the captain; bullets; the jet; …	
**Topic 433: **family; couple; mary; sarah; thomas; elizabeth; margaret; jesus; jane; matt; martin; daniel; frank; anna; nancy; …	
**Topic 454: **game; operation; experiment; treatment; procedure; scenario; victim; exercise: measurement; error; idea; …	
**Topic 525: **bush; the president; president bush; jesus; paul; the minister; clinton; smith; george w. bush; obama; …	
**Topic 561: **the world; christians; mulims; no matter; americans; jews; catholics; normoms; the chinese; hindus; …	
**Topic 570: **the sun; light; the moon; the beam; earth; mars; venus; laser; darkness; stars; jupiter; a hush; radiation; …	

**(b):** 

Relations	Relationship of topics (Topic_*i*_-Topic_*j*_)
Be cite	525–561; 251–286; 286–251; 251-251; 542–251; 371–286; 542–371; 542–286; 251–162; 134–286; 162–286; 371–251; 286–162; 286–171; 542–454; 286–538; 454–286; 286–10; 134–24; 538–286; 285-286; 575–454; 572–286; 328–286; 19–454; …

Blame on	116–428; 329–531; 116–531; 329-329; 329–116; 116–584; 329–584; 584–531; 314–531; 116–329; 480–531; 171–116; 116–160; 239–584; 458–531; 404–531; 584–116; 196–116; 531–458; 584-584; 531–116; 196–531; 176–531; 545–147; 171–2; …

Crash into	428–287; 428–571 390–106; 428–139; 428–390; 428-428; 390–139; 390-390; 390–287; 390–428; 428–570; 390–570; 139–106; 139–428; 139-139; 428–328; 287–106; 139–390; 390–328; 139–287; 428–374; 390–374; 287–139; 570–287; 106–428; …

Spot in	114–433; 433-433; 116–525; 114–287; 287–433; 114–570; 405–433; 433–405; 251–433; 114-114; 223–433; 570–433; 433–570; 114–132; 287–405; 114–251; 543–433; 230–433; 223–570; 114–424; 433–287; 433–114; 570-570; 433–132; 223–279; …

**Table 4:** Experimental datasets.

Category	Description	Number of tweets	Checked
Event 1	Death of Amy Winehouse	774	✓
Event 2	Space shuttle Atlantis lands safely, ending NASA's space shuttle program	45	
Event 3	Betty Ford dies	8	
Event 4	Richard Bowes, victim of London riots, dies in hospital	27	
Event 5	Flight Noar Linhas Aereas 4896 crashes, all 16 passengers dead	9	
Event 6	S&P downgrades US credit rating	275	✓
Event 7	US increases debt ceiling	73	✓
Event 8	Terrorist attack in Delhi	40	
Event 9	Earthquake in Virginia	271	✓
Event 10	Trevor Ellis (first victim of London riots) dies	63	
Event 11	Goran Hadzic, Yugoslavian war criminal, arrested	2	
Event 12	India and Bangladesh sign a peace pact	3	
Event 13	Plane carrying Russian hockey team Lokomotiv crashes, 44 dead	225	✓
Event 14	Explosion in French nuclear power plant Marcoule	137	✓
Event 15	NASA announces discovery of water on Mars	110	✓
Event 16	Google announces plans to buy Motorola Mobility	130	✓
Event 17	Car bomb explodes in Oslo, Norway	21	
Event 18	Gunman opens fire in children's camp on Utoya island, Norway	28	
Event 19	First artificial organ transplant	16	
Event 20	Petrol pipeline explosion in Kenya	27	
Event 21	Famine declared in Somalia	71	✓
Event 22	South Sudan declares independence	26	
Event 23	South Sudan becomes a UN member state	7	
Event 24	Three men die in riots in Birmingham	12	
Event 25	Riots break out in Tottenham	19	
Event 26	Rebels capture Tripoli international airport, Libya	4	
Event 27	Ferry sinks in Zanzibar, around 200 dead	21	

**Table 5:** Sample of similarities calculated by the proposed methods and the tf-*i*df method.

Tweets	tf-*i*df	tf-*i*df + ConceptNet	tf-*i*df + LDA-SP
T_1_: Amy Winehouse has passed away aged 27. T_2_: Amy Winehouse found death at her home in North London.	0.16	0.365	0.4

T_1_: Amy Winehouse has passed away aged 27. T_3_: Nelson Mandela, who led the peaceful transition from white-only rule, has died aged 95.	0.123	0.078	0.084

T_2_: Amy Winehouse found death at her home in North London. T_4_: plane crash kills majority of KHL team Lokomotiv.	0	0	0

T_4_: plane crash kills majority of KHL team Lokomotiv. T_5_: plane crash in Russia kills 36 or 37 assumed to be hockey player.	0.433	0.452	0.468

T_5_: plane crash in Russia kills 36 or 37 assumed to be hockey player. T_6_: plane crash, helicopter, was in Moscow with 2 dead.	0.272	0.146	0.104

**Table 6:** Experimental results.

Category	*k*NN			SVM			*k*NN-M1 (ours)			*k*NN-M2 (ours)		
	*P*	*R*	*F*	*P*	*R*	*F*	*P*	*R*	*F*	*P*	*R*	*F*
Event 1	76.3	71.6	73.8	75.2	75.5	75.3	86.1	77.5	81.6	88.2	82.6	**85.3**
Event 6	84.6	85.4	84.9	86.9	87.2	87.1	91.1	89.4	**90.2**	89.1	86.4	87.7
Event 7	78.9	72.3	75.5	80.4	76.2	78.2	87.5	82.3	**84.8**	82.4	78.9	80.6
Event 9	83.9	78.8	81.3	85.5	80.2	82.3	93.8	92.9	**93.4**	87.2	83.3	85.2
Event 13	83.6	72.4	77.5	82.8	75.6	79.1	86.2	80.5	83.3	87.3	82.6	**84.9**
Event 14	70.1	67.8	68.9	71.6	70.0	70.8	85.2	78.7	**81.8**	83.8	74.3	78.8
Event 15	79.3	71.5	75.2	81.0	70.8	75.6	90.1	87.9	**88.9**	88.8	85.8	87.3
Event 16	80.5	72.4	76.2	82.5	73.1	77.5	85.7	80.0	**82.8**	85.5	79.6	82.5
Event 21	81.6	74.1	77.7	82.4	76.8	79.5	83.9	77.8	80.7	85.4	77.1	**81.0**
Overall	79.5	74.4	76.8	79.9	77.0	78.4	87.9	82.4	**85.0**	87.4	82.3	84.7
